# Chronic Mild Cold Conditioning Modulates the Expression of Hypothalamic Neuropeptide and Intermediary Metabolic-Related Genes and Improves Growth Performances in Young Chicks

**DOI:** 10.1371/journal.pone.0142319

**Published:** 2015-11-16

**Authors:** Phuong Nguyen, Elizabeth Greene, Peter Ishola, Geraldine Huff, Annie Donoghue, Walter Bottje, Sami Dridi

**Affiliations:** 1 Center of Excellence for Poultry Science, University of Arkansas, Fayetteville, AR, 72701, United States of America; 2 USDA, Agricultural Research Service, Poultry Production and Product Safety Research Unit, Fayetteville, AR, 72701, United States of America; University of California, Davis, UNITED STATES

## Abstract

**Background:**

Low environmental temperatures are among the most challenging stressors in poultry industries. Although landmark studies using acute severe cold exposure have been conducted, still the molecular mechanisms underlying cold-stress responses in birds are not completely defined. In the present study we determine the effect of chronic mild cold conditioning (CMCC) on growth performances and on the expression of key metabolic-related genes in three metabolically important tissues: brain (main site for feed intake control), liver (main site for lipogenesis) and muscle (main site for thermogenesis).

**Methods:**

80 one-day old male broiler chicks were divided into two weight-matched groups and maintained in two different temperature floor pen rooms (40 birds/room). The temperature of control room was 32°C, while the cold room temperature started at 26.7°C and gradually reduced every day (1°C/day) to reach 19.7°C at the seventh day of the experiment. At day 7, growth performances were recorded (from all birds) and blood samples and tissues were collected (n = 10). The rest of birds were maintained at the same standard environmental condition for two more weeks and growth performances were measured.

**Results:**

Although feed intake remained unchanged, body weight gain was significantly increased in CMCC compared to the control chicks resulting in a significant low feed conversion ratio (FCR). Circulating cholesterol and creatine kinase levels were higher in CMCC chicks compared to the control group (*P*<0.05). CMCC significantly decreased the expression of both the hypothalamic orexigenic neuropeptide Y (NPY) and anorexigenic cocaine and amphetamine regulated transcript (CART) in chick brain which may explain the similar feed intake between the two groups. Compared to the control condition, CMCC increased the mRNA abundance of AMPKα1/α2 and decreased mTOR gene expression (*P*<0.05), the master energy and nutrient sensors, respectively. It also significantly decreased the expression of fatty acid synthase (FAS) gene in chick brain compared to the control. Although their roles are still unknown in avian species, adiponectin (Adpn) and its related receptors (AdipoR1 and 2) were down regulated in the brain of CMCC compared to control chicks (*P*<0.05). In the liver, CMCC significantly down regulated the expression of lipogenic genes namely FAS, acetyl-CoA carboxylase alpha (ACCα) and malic enzyme (ME) and their related transcription factors sterol regulatory element binding protein 1/2 (SREBP-1 and 2). Hepatic mTOR mRNA levels and phosphorylated mTOR at Ser^2448^ were down regulated (*P*<0.05), however phosphorylated ACCα^Ser79^ (inactivation) was up regulated (P<0.05) in CMCC compared to control chicks, indicating that CMCC switch hepatic catabolism on and inhibits hepatic lipogenesis. In the muscle however, CMCC significantly up regulated the expression of carnitine palmitoyltransferase 1 (CPT-1) gene and the mRNA and phosphorylated protein levels of mTOR compared to the control chicks, indicating that CMCC enhanced muscle fatty acid β-oxidation.

**Conclusions:**

In conclusion, this is the first report indicating that CMCC may regulate AMPK-mTOR expression in a tissue specific manner and identifying AMPK-mTOR as a potential molecular signature that controls cellular fatty acid utilization (inhibition of hepatic lipogenesis and induction of muscle fatty acid β-oxidation) to enhance growth performance during mild cold acclimation.

## Introduction

Global warming can lead to extreme weather in various portions of the globe so that some regions have extreme snowfall and others have increased intense and frequent heat waves [1]. These climate changes are predicted to continue to rise [2]. Environmental stressors (cold and heat) are already affecting animals, insects and crops [3]. In expanding worldwide broiler (meat-type chicken) production, the effects of cold stress on growth performances are controversial depending on the stress severity, the exposure period, and the age of the chicken. Indeed, sudden severe decrease of environmental temperature has been reported to negatively affect growth performance (feed efficiency, meat yield, ascites, mortality) [4] and result in serious annual economic losses to the livestock and poultry industries [5]. On the other hand, conditioning exposure of chickens at an early age to moderate decreased environmental temperature has been reported to have a long-lasting effect and improve the ability of the chicken to acclimatize and to cope better with stressors in later life [6]. Thus, insights into the molecular mechanisms by which cold exposure affects chicken metabolism are of uppermost interest in animal biology, health, wellbeing, and feed efficiency improvement.

Cold stress induces many cellular alterations that in turn lead to various neuroendocrine, physiological, and immunological adaptations [7]. Animals living in extreme climates show a marked seasonal variation in both energy homeostasis (energy intake and expenditure) and intermediary metabolism as a response to the changing metabolic requirements imposed by differences in environmental temperature [8]. These variables are mediated by complex molecular networks that are still not completely defined.

Depending on the type, degree and duration of the stress, cells can develop highly efficient stress response and protein quality control systems to ensure their survival or activate stress signaling cascades that proceed into cell-death pathway. Stress rapidly initiates the increased synthesis of a group of stress proteins belonging to the heat shock protein (HSP) families. These ubiquitously expressed molecular chaperones are classified into about six families (HSP-10 to HSP-100) on the basis of their monomeric molecular weight [9]. They carry out crucial housekeeping functions and orchestrate folding/unfolding and assembly/disassembly of protein complexes to maintain normal cell function. HSPs are regulated at transcriptional levels through HSP transcription factors (HSF1-4) that bind to heat shock response element (HSE) in the upstream promoter regions of HSPs [10]. Additionally, HSPs are subjected to various post-translational modifications such as acetylation, S-nitrosylation and glycosylation [11–13].

Emerging evidence indicates that the regulation of energy homeostasis and the stress response are coupled physiological processes [14]. We and other groups have previously shown that HSP-70 gene expression is regulated by various feeding-related hormones [15–18]. We also showed that acute cold stress alters the expression of key genes (leptin and uncoupling protein, UCP) involved in the regulation of energy intake and expenditure in 5 week old broiler chickens. Several recent studies investigating the effect of acute or chronic severe cold exposure (-15 to 12°C) on neuropeptides, antioxidant, immune, immunoglobulin and cytokine systems in immune organs, heart, hypothalamus and gastrointestinal tract in old (15 days to 4 weeks) chickens have been reported [5,19–24]. However, data related to the effect of chronic mild cold conditioning (CMCC) on metabolic-related genes in young chicks are scarce.

Therefore, the present study was designed to determine the effects of CMCC on the expression of hypothalamic feeding-related neuropeptides, hepatic lipogenesis- and muscle energy expenditure-related genes, and the expression of HSPs and their related transcription factors HSFs in three metabolically important tissues: brain (the main site of feed intake control), liver (the main site of lipogenesis), and muscle (the main site for thermogenesis and energy expenditure) in young chicks.

## Materials and Methods

### Ethic Statement

The present study was conducted in accordance with the recommendations in the guide for the care and use of laboratory animals of the National Institutes of Health and the protocol was approved by the University of Arkansas Animal Care and Use Committee under protocol 13026.

### Animals

Male broiler chicks (Cobb males from the Cobb 500 female line) were hatched from a single flock of 29-week-old hens in their third week of lay. At day one post-hatch, chicks (n = 80) were weighed, divided in four body weight-matched groups and placed into four randomly assigned floor pens (20 birds/pen) containing fresh pine shavings in two separate environmentally controlled rooms. Control groups were maintained at 32°C for the first week. Cold-stressed groups were maintained at 26.7°C for the first day and the ambient temperature was reduced gradually by 1°C every day to reach 19.7°C at the seventh day. Chicks were given *ad libitum* access to clean water and a complete starter diet (12.7 MJ metabolizable energy Kg^-1^ and 220 g crude protein Kg^-1^). A relative humidity of ∼ 55% and a 23 h light/1h dark cycles were maintained until the end of the experiment (7 days). Body weight and feed intake were recorded weekly. Body temperature was measured daily using a Braun Thermoscan IRT4520 thermometer (Kaz Inc, Southborough, MA). EDTA-treated whole blood and serum were collected and assayed for standard hematology and metabolites. Birds were cervically dislocated and whole brain, liver and leg muscle tissues were collected, snap frozen in liquid nitrogen and stored at -80°C until use.

### Measurement of circulating metabolites

Commercial colorimetric diagnostic kits were used to measure plasma glucose (Ciba Corning Diagnostics Corp., OH), triglycerides, cholesterol and creatine kinase (Chiron Diagnostics, Cergy Pontoise, France), lactate dehydrogenase (LDH, Bayer Healthcare, Dublin, Ireland), and uric acid levels (Pointe Scientific Inc, Canton, MI) with an automated spectrophotometer according to manufacturer’s recommendations. Briefly, glucose concentration was estimated by the formation of NADH at 340 nm absorbance. When phosphorylated with ATP and hexokinase, glucose yields glucose-6- phosphate and ADP which, in turn, is catalyzed by G-6-PD to form 6-phosphogluconate and NADH. NADH formation is directly proportional to the amount of glucose in the sample. Triglyceride levels were enzymatically determined based on the action of lipase, glycerol kinase and glycerol-phosphate oxidase at 540 nm. Cholesterol levels were enzymatically estimated in the presence of cholesterol esterase, cholesterol oxidase and peroxidase at 500 nm. Creatine kinase levels were determined based on the rate of NADPH formation measured at 340 nm in the presence of HK and G-6-PD. LDH activity was estimated following the oxidation of lactate to pyruvate at 340 nm. Uric acid levels were measured using the coupling of 4-aminoantipyrine with 2-hydroxy-2,4,6-tribromobenzoic acid and hydrogen peroxide in the presence of peroxidase at 520 nm.

### RNA isolation, reverse transcription and quantitative real-time PCR

Total RNA was extracted from chick tissues by Trizol reagent (Life Technologies) according to manufacturer’s recommendations, DNAse treated and reverse transcribed (Quanta Biosciences). RNA integrity and quality was assessed using 1% agarose gel electrophoresis and RNA concentrations and purity were determined for each sample by Take 3 micro volume plate using Synergy HT multi-mode microplate reader (BioTek). The RT products (cDNAs) were amplified by real-time quantitative PCR (Applied Biosystems 7500 Real-Time PCR system) with Power SYBR green Master Mix (Life Technologies). Oligonucleotide primers used for chicken hypothalamic neuropeptides, HSPs, HSFs, lipogenesis- and energy expenditure-related genes are summarized in Table 1. The qPCR cycling conditions were 50°C for 2 min, 95°C for 10 min followed by 40 cycles of a two-step amplification program (95°C for 15 s and 58°C for 1 min). At the end of the amplification, melting curve analysis was applied using the dissociation protocol from the Sequence Detection system to exclude contamination with unspecific PCR products. The PCR products were also confirmed by agarose gel and showed only one specific band of the predicted size. For negative controls, no RT products were used as templates in the qPCR and verified by the absence of gel-detected bands. Relative expressions of target genes were determined by the 2^–ΔΔCt^ method [25].

**Table 1:** Oligonucleotide qPCR primers.

Gene	Accession number^a^	Primer sequence (5’ → 3’)	Orientation	Product size (bp)
***NPY***	**NM_205473**	CATGCAGGGCACCATGAG	Forward	55
		CAGCGACAAGGCGAAAGTC	Reverse	
***AgRP***	**AB029443**	GCGGGAGCTTTCACAGAACA	Forward	58
		CGACAGGATTGACCCCAAAA	Reverse	
***Ox***	**AB056748**	CCAGGAGCACGCTGAGAAG	Forward	67
		CCCATCTCAGTAAAAGCTCTTTGC	Reverse	
***Ox1R***	**AB110634**	TGCGCTACCTCTGGAAGGA	Forward	58
		GCGATCAGCGCCCATTC	Reverse	
***Ox2R***	**XM_004945362**	AAGTGCTGAAGCAACCATTGC	Forward	61
		AAGGCCACACTCTCCCTTCTG	Reverse	
***Adpn***	**AY786316**	ATGGACAAAAGGGAGACAAAGG	Forward	64
		TCCAGCACCCATATACCCAAA	Reverse	
***AdipoR1***	**NM_001031027**	CCGGGCAAATTCGACATC	Forward	58
		CCACCACGAGCACATGGA	Reverse	
***AdipoR2***	**NM_001007854**	TTGCCACTCGGAAGGTGTTT	Forward	60
		AGTGCAATGCCAGAATAATCCA	Reverse	
***Pomc***	**AB019555**	GCCAGACCCCGCTGATG	Forward	56
		CTTGTAGGCGCTTTTGACGAT	Reverse	
***Cart***	**KC249966**	GCTGGAGAAGCTGAAGAGCAA	Forward	60
		GGCACCTGCCCGAACTT	Reverse	
***Ob-R***	**NM_204323**	GCAAGACCCTCTCCCTTATCTCT	Forward	70
		TCTGTGAAAGCATCATCCTGATCT	Reverse	
***Ghrl***	**AY303688**	CACTCCTGCTCACATACAAGTTCA	Forward	75
		TCATATGTACACCTGTGGCAGAAA	Reverse	
***GHS-R1a***	**NM_204394**	GCACAAATCGGCAAGGAAA	Forward	61
		GTGACATCTCCCAGCAAATCC	Reverse	
***CRH***	**NM_001123031**	TCAGCACCAGAGCCATCACA	Forward	74
		GCTCTATAAAAATAAAGAGGTGACATCAGA	Reverse	
***FAS***	**JO3860**	ACTGTGGGCTCCAAATCTTCA	Forward	70
		CAAGGAGCCATCGTGTAAAGC	Reverse	
***ACCα***	**NM_205505**	CAGGTATCGCATCACTATAGGTAACAA	Forward	74
		GTGAGCGCAGAATAGAAGGATCA	Reverse	
***SCD-1***	**NM_204890**	CAATGCCACCTGGCTAGTGA	Forward	52
		CGGCCGATTGCCAAAC	Reverse	
***AMPKα1***	**NM_001039603**	CCACCCCTGTACCGGAAATA	Forward	68
		GGAAGCGAGTGCCAGAGTTC	Reverse	
***AMPKα2***	**NM_001039605**	GCGGAGAGAATCTGCTGGAA	Forward	62
		TGTAAGCATGGACGTGTTGAAGA	Reverse	
***AMPKβ1***	**NM_001039912**	TTGGCAGCAGGATCTGGAA	Forward	60
		AAGACTGTTGGTCGAGCTTGAGT	Reverse	
***AMPKβ2***	**NM_001044662**	TGTGACCCGGCCCTACTG	Forward	56
		GCGTAGAGGTGATTGAGCATGA	Reverse	
***AMPKγ1***	**NM_001034827**	CAAGCCGTTGGTCTGCATCT	Forward	56
		GGGAGGAGACGGCATCAA	Reverse	
***AMPKγ2***	**NM_001278142**	TGCCATGCCATTCTTGGA	Forward	62
		CCACCTTGCGAGAAGCATTT	Reverse	
***AMPKγ3***	**NM_001031258**	CCCAAGCCACGCTTCCTA	Forward	57
		ACGGAAGGTGCCGACACA	Reverse	
***mTOR***	**XM_417614**	CATGTCAGGCACTGTGTCTATTCTC	Forward	77
		CTTTCGCCCTTGTTTCTTCACT	Reverse	
***ME***	**AF408407**	AGATGAAGCTGTCAAAAGGATATGG	Forward	62
		CACGCCCCTTCACTATCGA	Reverse	
***ATPcl***	**NM_001030540**	CTTTTAAGGGCATTGTTAGAGCAAT	Forward	65
		CCTCACCTCGTGCTCTTTCAG	Reverse	
***SREBP-1***	**AY029224**	CATCCATCAACGACAAGATCGT	Forward	82
		CTCAGGATCGCCGACTTGTT	Reverse	
***SREBP-2***	**AJ414379**	GCCTCTGATTCGGGATCACA	Forward	63
		GCTTCCTGGCTCTGAATCAATG	Reverse	
***INSIG-1***	**NM_001030966**	TGGCGCTGGTGCTGAAC	Forward	63
		TGACCTCGTCGGGAAACAG	Reverse	
***INSIG-2***	**NM_001031261**	CAGCGCTAAAGTGGATTTTGC	Forward	65
		CAATTGACAGGGCTGCTAACG	Reverse	
***UCP***	**NM_204107**	TGGCAGCGAAGCGTCAT	Forward	59
		TGGGATGCTGCGTCCTATG	Reverse	
***ANT***	**AB088686**	GCAGCTGATGTCGGCAAA	Forward	56
		CAGTCCCCGAGACCAGAGAA	Reverse	
***NRF-1***	**NM_001030646**	GGCCAACGTCCGAAGTGAT	Forward	55
		CCATGACACCCGCTGCTT	Reverse	
***Ski***	**M28517**	GGCCCTGCTGCTTTCTCA	Forward	75
		AGGTTCCGCTGGGTCTTTG	Reverse	
***CPT-1***	**AY675193**	GCCCTGATGCCTTCATTCAA	Forward	60
		ATTTTCCCATGTCTCGGTAGTGA	Reverse	
***S6K1***	**NM_001109771**	GTCAGACATCACTTGGGTAGAGAAAG	Forward	60
		ACGCCCTCGCCCTTGT	Reverse	
***PGC-1α***	**NM_001006457**	GAGGATGGATTGCCTTCATTTG	Forward	62
		GCGTCATGTTCATTGGTCACA	Reverse	
***PGC-1β***	**XM_414479**	TTGCCGGCATTGGTTTCT	Forward	66
		CACGGGAAGCCACAGGAA	Reverse	
***PPARα***	**AF163809**	CAAACCAACCATCCTGACGAT	Forward	64
		GGAGGTCAGCCATTTTTTGGA	Reverse	
***PPARδ***	**NM_001001460**	CACTGCAGGAACAGAACAAAGAA	Forward	67
		TCCACAGAGCGAAACTGACATC	Reverse	
***HSP70***	**JO2579**	GGGAGAGGGTTGGGCTAGAG	Forward	55
		TTGCCTCCTGCCCAATCA	Reverse	
***HSP60***	**NM_001012916**	CGCAGACATGCTCCGTTTG	Forward	55
		TCTGGACACCGGCCTGAT	Reverse	
***HSP27***	**XM_001231557**	TTGAAGGCTGGCTCCTGATC	Forward	58
		AAGCCATGCTCATCCATCCT	Reverse	
***HSF1***	**L06098**	GAGACGGACCCGCTGATCT	Forward	58
		GGTCGAACACATGGAAGCTGTT	Reverse	
***HSF2***	**NM_001167764**	GCCCAGCAACCAGCTTATCA	Forward	63
		TGTTCATCCAACACCAAGAAACTC	Reverse	
***HSF3***	**L06126**	CAGAGCGACGACGTCATCTG	Forward	66
		CCGCTGCTCATCCAGGAT	Reverse	
***HSF4***	**NM_001172374**	CAAAGAGGTGCTGCCCAAGT	Forward	60
		AGCTGCCGGACGAAACTG	Reverse	
***18S***	**AF173612**	TCCCCTCCCGTTACTTGGAT	Forward	60
		GCGCTCGTCGGCATGTA	Reverse	

^a^ Accession number refer to Genbank (NCBI).

ACC, acetyl-CoA carboxylase; AdipoR, adiponectin receptor; Adpn, adiponectin; AgRP, agouti related peptide; AMPK, AMP-activated protein kinase; ANT, adenine nucleotide translocator; ATPcl, ATP citrate lyase; Cart, cocaine and amphetamine regulated transcript; CPT, carnitine palmitoyltransferase; CRH, corticotropin releasing hormone; FAS, fatty acid synthase; Ghrl, ghrelin; GHS-R1a, ghrelin receptor; HSF, heat shock factor; HSP, heat shock protein; INSIG, insulin induced gene; ME, malic enzyme; mTOR, mechanistic target of rapamycin; NPY, neuropeptide Y; NRF, nuclear respiratory factor; Ob-R, leptin receptor; Ox, orexin; OxR, orexin receptor; PGC, PPARδ coactivator; Pomc, pro-opiomelanocortin; PPAR, peroxisome proliferator activator of transcription; SCD, stearoyl-CoA desaturase; Ski, avian sarcoma viral oncogene homolog; S6K1, ribosomal S6 kinase; SREBP, sterol regulatory element binding protein; UCP, uncoupling protein.

### Western blot analysis

Total proteins were extracted from chick tissues, quantified, and subjected to Western blot as we previously described [26,27]. The rabbit polyclonal anti-phospho mechanistic target of rapamycin (mTOR)^Ser2448^ (#2971), anti-mTOR (#2972), anti-phospho AMP-activated protein kinase alpha (AMPKα)^Thr172^ (#2531), anti-AMPKα1 (#2795), anti-AMPKα2 (#2757) anti-phospho acetyl-CoA carboxylase alpha (ACCα)^Ser79^ (#3661), anti-ACCα (#3662), anti-HSP90 (#PA5-17610), and mouse monoclonal anti-HSP70 (#MAI-91159) were used. Protein loading was assessed by immunoblotting with the use of rabbit anti-β actin (#4967) or rabbit anti-vinculin (#V4139). Pre-stained molecular weight marker (Precision Plus Protein Dual Color) was used as a standard (BioRad). All primary antibodies were purchased from Cell Signaling Technology, except for the anti-HSP70 and anti-HSP90 which were purchased from Pierce Thermo Scientific, and anti-vinculin which was purchased from Sigma-Aldrich. The secondary antibodies were used (1:5000) for 1 h at room temperature. The signal was visualized by enhanced chemiluminescence (ECL plus) (GE Healthcare Bio-Sciences) and captured by FluorChem M MultiFluor System (Proteinsimple). Image Acquisition and Analysis were performed by AlphaView software (Version 3.4.0, 1993–2011, Proteinsimple).

### Statistical analysis

Growth performance (feed intake FI, body weight gain BWG, and feed conversion ratio FCR), plasma metabolite parameters (cholesterol, glucose, triglyceride, uric acid, LDH and creatine kinase), and gene and protein expression data were analyzed by the Student’s unpaired *t*-test. Body temperature data were analyzed using two-way repeated-measures ANOVA with time as the repeated measure and treatment (TN vs cold exposure) as factors. Data are expressed as the mean ± SEM and analyzed using Graph Pad Prism software (version 6 for windows). Significance was set at *P*<0.05.

## Results

### CMCC improves growth performances in chicks

**Fig 1:**
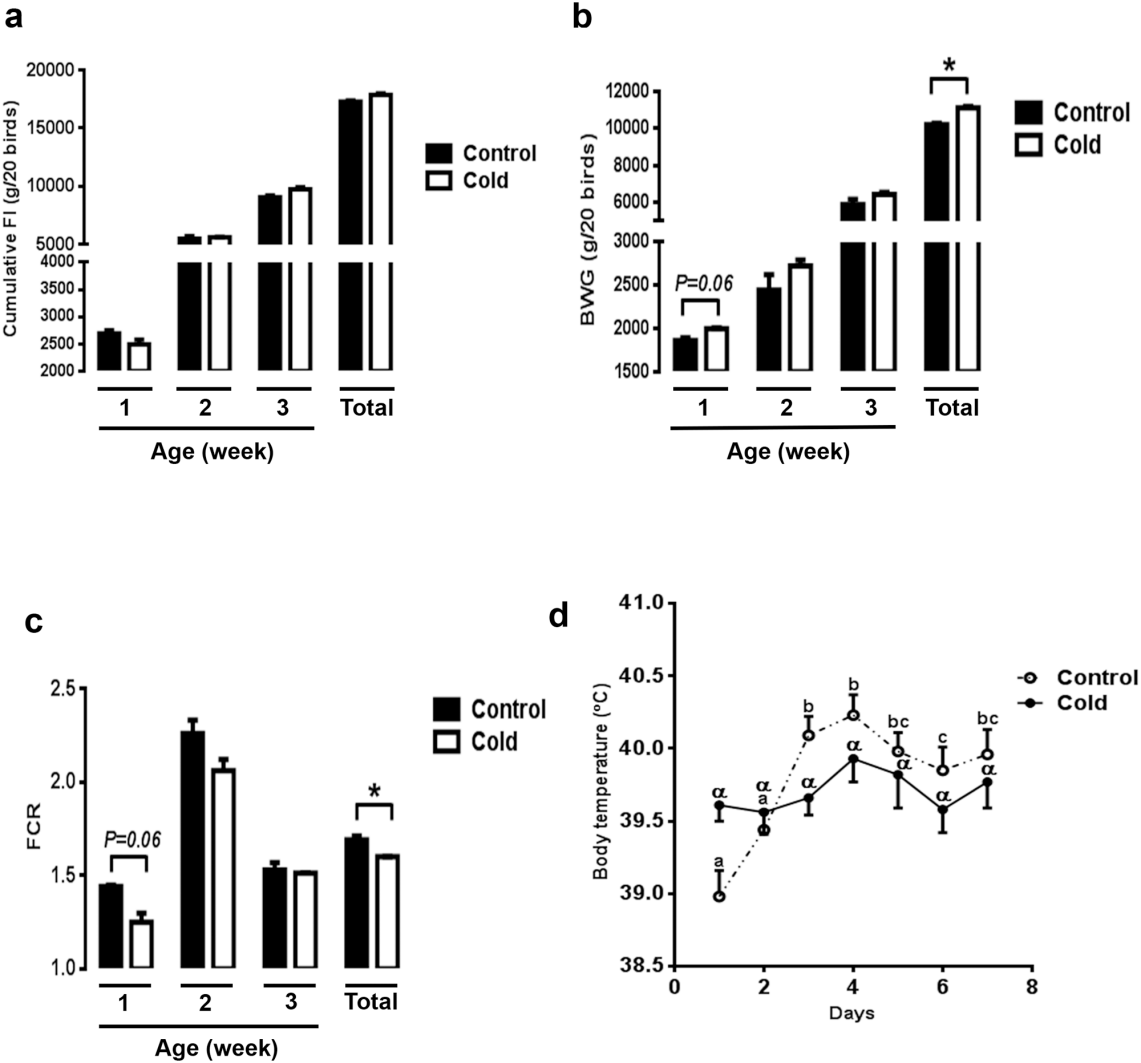
Effect of CMCC on growth performances in young broiler chicks. Cumulative feed intake FI (a), body weight gain BWG (b), feed conversion ratio FCR (c), and body temperature (d). Data are presented as mean ± SEM (n = 40) for each week and for the total experimental period (3 weeks). *P<0.05. Different letters indicate daily difference in body temperature within each group (a-c and α, difference within control and cold group, respectively).

As shown in Fig 1a, both weekly and total (whole 3-wk period) cumulative feed intake did not differ between the control and CMCC chicks, however the total body weight gain (3-wk period) was significantly higher in CMCC group compared to the control resulting in significant lower FCR (*P*<0.05, Fig 1b and 1c). The body temperature (BT) of the control chicks significantly increased from day 3 to day 7 of the first week compared to day 1, however the BT of CMCC chicks remained the same during the first 7 days (Fig 1d). When BT of both groups were plotted together, CMCC chicks exhibited higher BT at the first day and lower BT from day 3 to day 7; however the difference was not statistically discernable (Fig 1d).

### CMCC alters circulating metabolite levels

**Fig 2:**
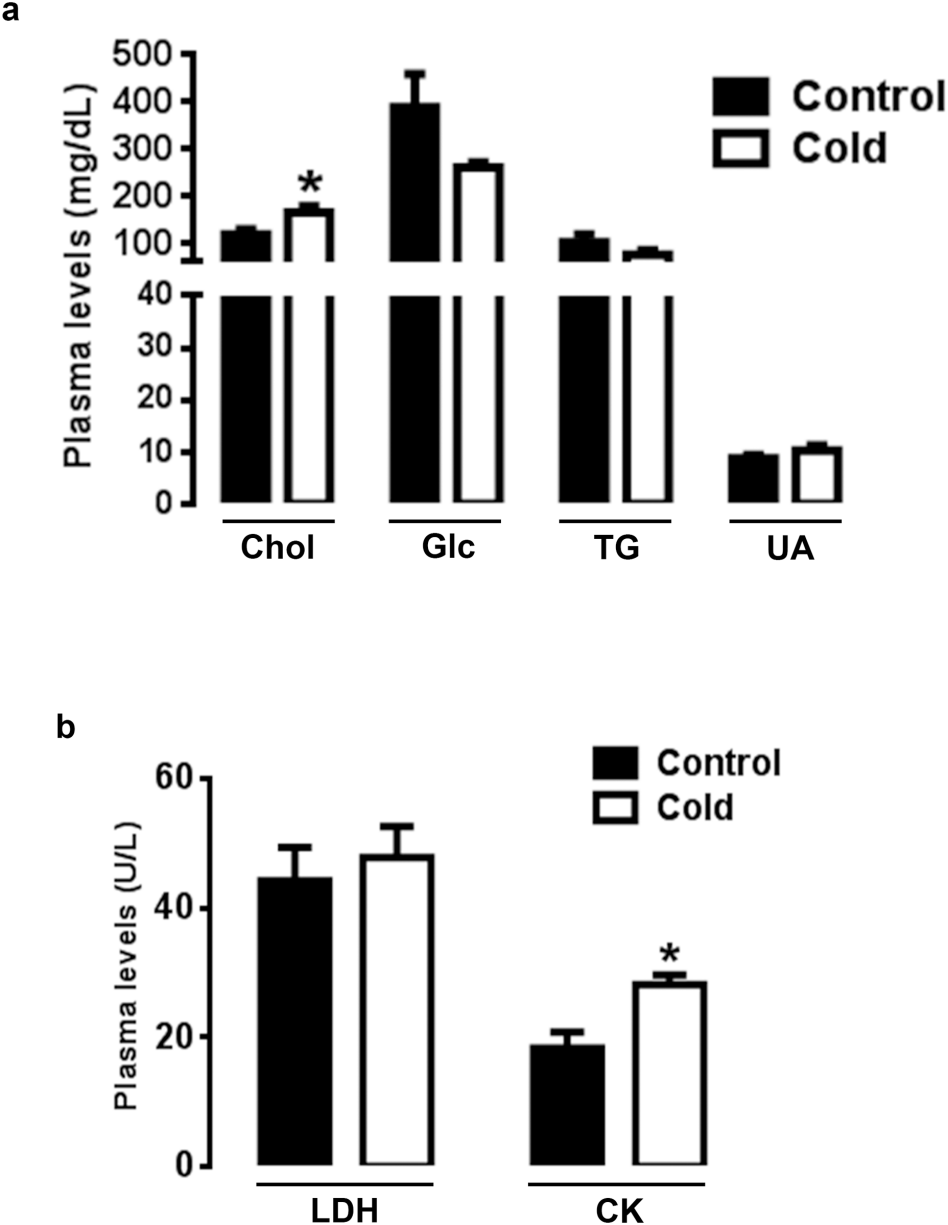
Effect of CMCC on plasma metabolite levels. Plasma levels of Chol, Glc, TG, UA (a), LDH and CK (b) were determined at the first week using commercial kits as described in materials and methods. Data are presented as mean ± SEM (n = 6). *Indicate a significant difference between cold and control group (*P*<0.05).

CMCC significantly increased the circulating levels of cholesterol (Chol) and creatine kinase (CK) without affecting circulating glucose (Glc), triglyceride (TG), uric acid (UA) and L-lactate dehydrogenase (LDH) levels (Fig 2a and 2b).

### CMCC affects the expression of hypothalamic feeding-related genes in chicks

**Fig 3:**
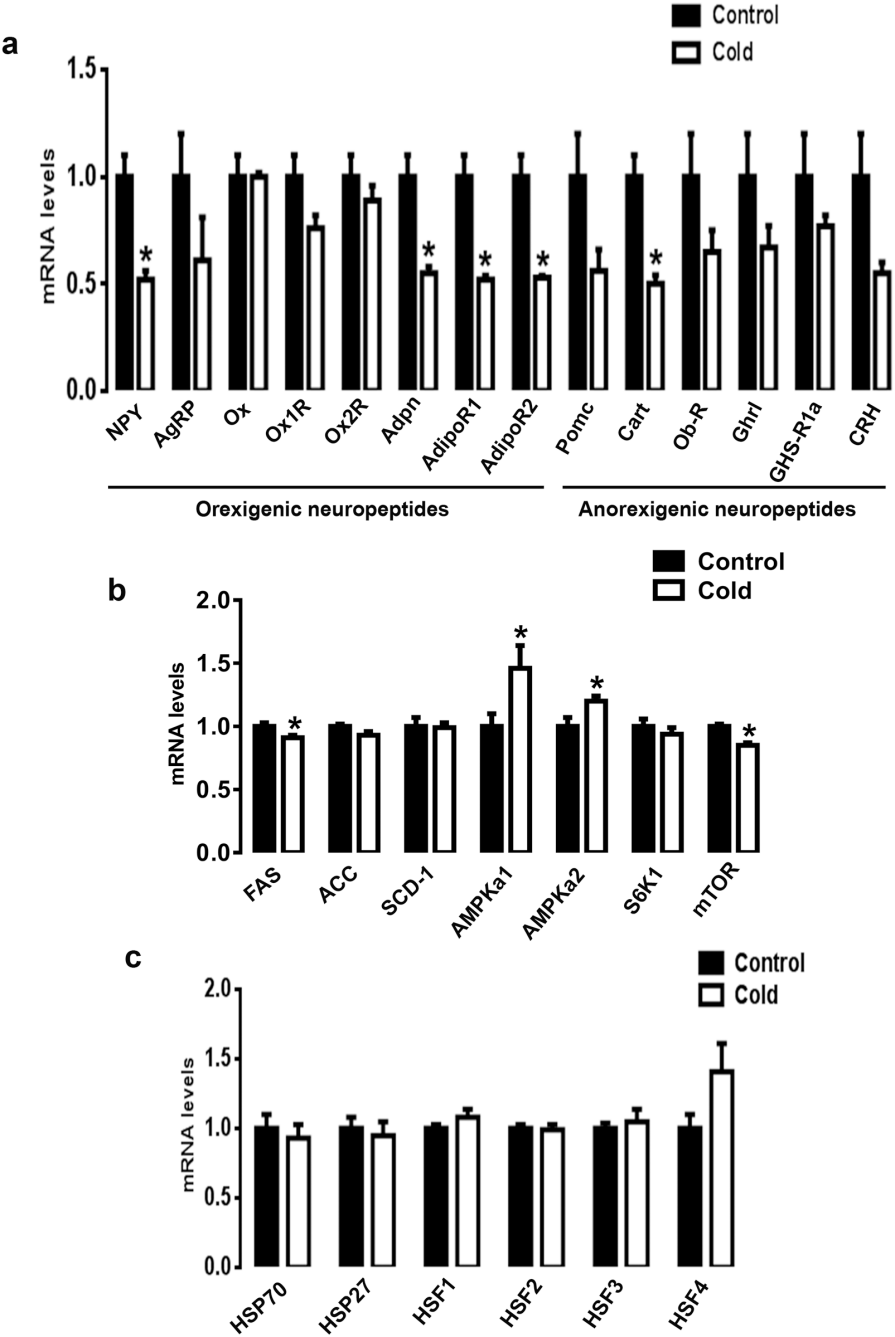
Effect of CMCC on feeding-related genes and HSPs in the brain of young broiler chicks. Relative expression of hypothalamic feeding-related neuropeptides (a), lipogenic genes (b), HSPs and HSF (c) were determined by qPCR using 2^-ΔΔCt^ method [25]. Data are presented as mean ± SEM (n = 6). * Indicate a significant difference between cold and control group (*P*<0.05).

Since the effect of some neuropeptides in chicken is not fully understood and established, we classified them in the present section based on their effects in mammals. CMCC significantly down-regulated the expression of the orexigenic hypothalamic neuropeptide NPY, adiponectin (*Adpn*) and its related receptors *AdipoR*1 and *AdipoR*2 compared to the control group (*P*<0.05, Fig 3a). The expression of hypothalamic agouti-related protein (*AgRP*), orexin (*Ox*) and its related receptors (*Ox1R* and *Ox2R*) remained unchanged between the control and CMCC groups (Fig 3a). Among the anorexigenic neuropeptides, only cocaine and amphetamine regulated transcript (*Cart*) was significantly down-regulated in CMCC compared to control chicks (*P*<0.05, Fig 3a). CMCC significantly down regulated the expression of hypothalamic fatty acid synthase (*FAS*) and mechanistic target of rapamycin (mTOR), and up regulated the expression of AMP-activated protein kinase alpha 1 and 2 (*AMPKα1 and α2*) (*P*<0.05, Fig 3b) and AMPKγ1/2 (*P*<0.05, S1 Fig). The expression of acetyl-CoA carboxylase alpha (ACCα), stearoyl-CoA desaturase1 (SCD-1), ribosomal p70 S6 kinase (*S6K1*), and AMPKβ1/β2 and γ3 remained unchanged between the cold and the control groups (Fig 3b and S1 Fig). Neither HSP (*HSP70* and *HSP27*), nor their related transcription factors (*HSF1-4*) mRNA abundances, were affected by CMCC compared to control group (Fig 3c).

### CMCC down-regulates hepatic lipogenic gene expression in chicks

**Fig 4:**
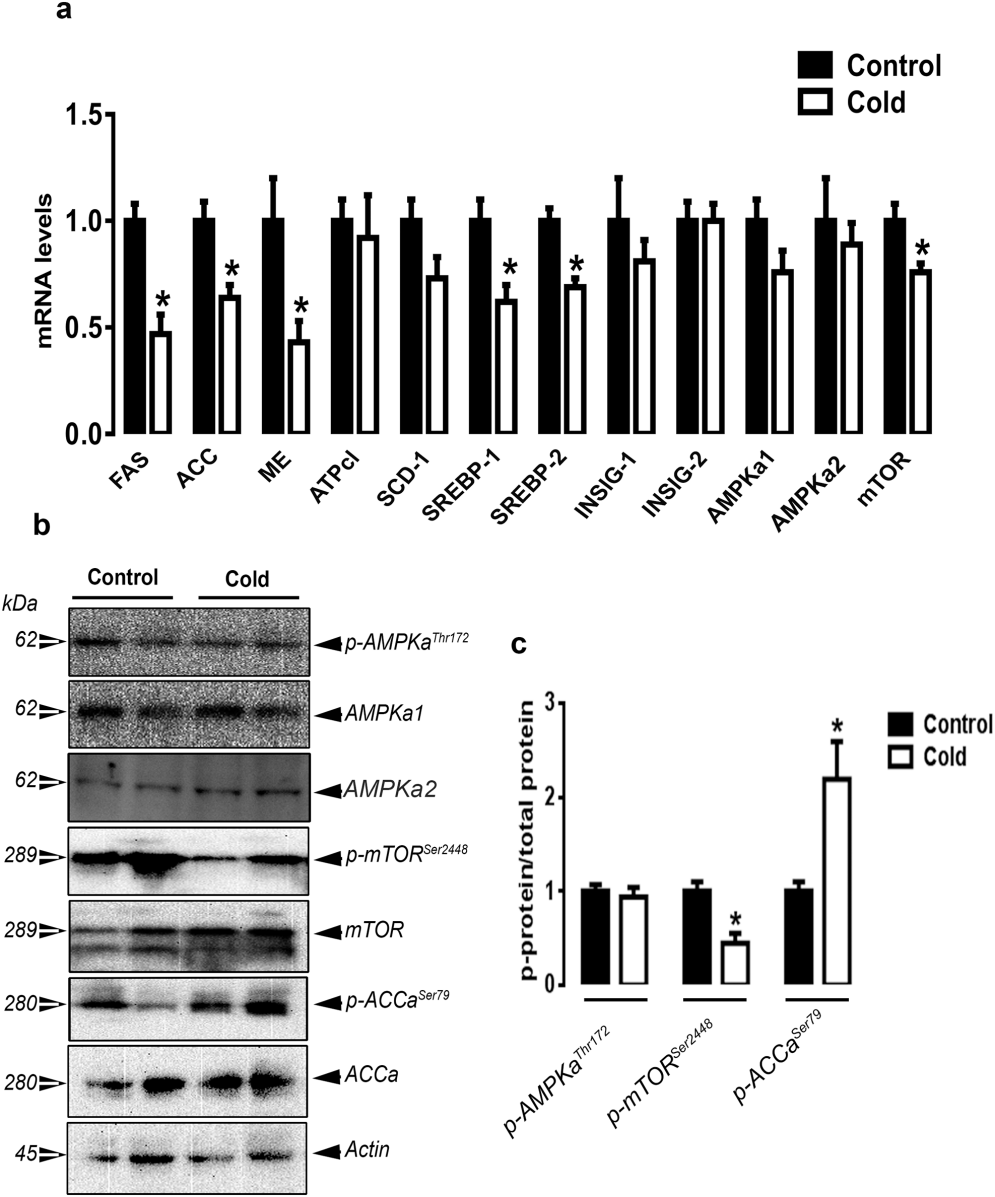
Effect of CMCC on lipogenesis-related genes in liver of young broiler chicks. Relative expression of lipogenic genes and their related transcription factors (a) was measured by qPCR as described in material and methods. Phosphorylated and total protein levels of AMPK, mTOR and ACC were determined by Western blot (b) and presented as p-protein/total protein ratio (c). β-actin was used as loading and housekeeping control. Data are presented as mean ± SEM (n = 6). *Indicate a significant difference between cold and control group (*P*<0.05).

**Fig 5:**
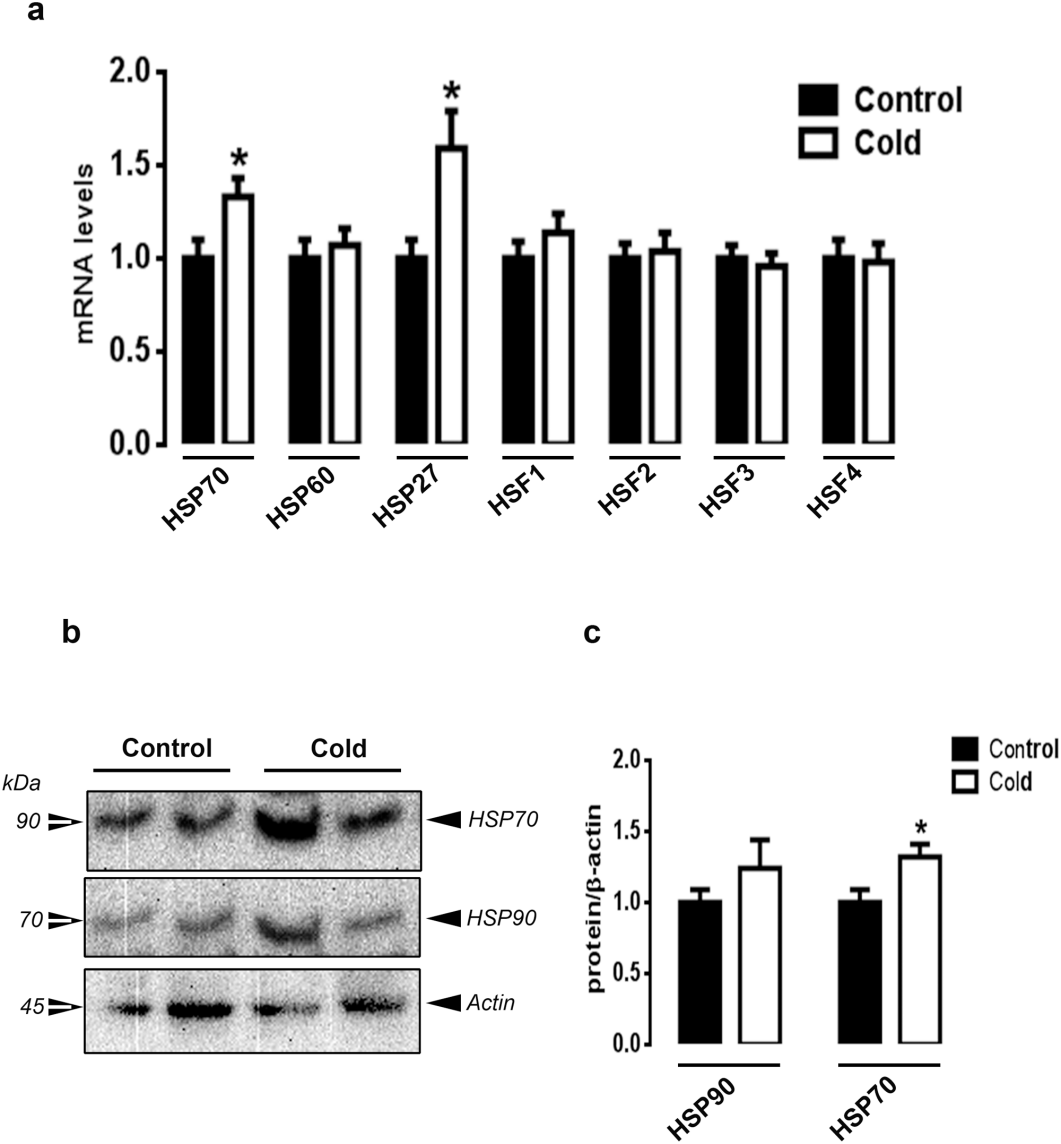
Effect of CMCC on HSP and HSF expression in liver of young broiler chicks. HSP and HSF mRNA levels were measured by qPCR (a). HSP70 and HSP90 protein levels were determined by Western blot (b) and presented as normalized ratio to β-actin (c). The values represent the mean ± SEM (n = 6). *Indicates a significant difference between cold and control group (*P*<0.05).

CMCC significantly down-regulated the expression of hepatic FAS, ACCα, malic enzyme (ME), sterol regulatory element binding protein 1 and 2 (SREBP-1 and 2) and mTOR compared to the control group (P<0.05, Fig 4a). The expression of ATP citrate lyase (ATPcl), stearoyl-CoA desaturase 1 (SCD-1), AMPKα1/2, AMPKβ1/2, AMPKγ1/2/3, and insulin induced gene 1 and 2 (INSIG-1 and 2) did not differ between the control and CMCC groups (Fig 4a and S1 Fig). Concomitant with these changes, CMCC decreased the phosphorylated levels of mTOR^Ser2448^ and increased the phosphorylated levels of ACCα^Ser79^ compared to the control group (Fig 4b and 4c). CMCC induced the hepatic HSP27 and HSP70 mRNA levels and HSP70 protein expression compared to the control group (*P*<0.05, Fig 5b and 5c), however hepatic expression of HSF1-4 remained unchanged between the two groups (Fig 5a).

### CMCC alters the expression of metabolic-related genes in young chick muscles

**Fig 6:**
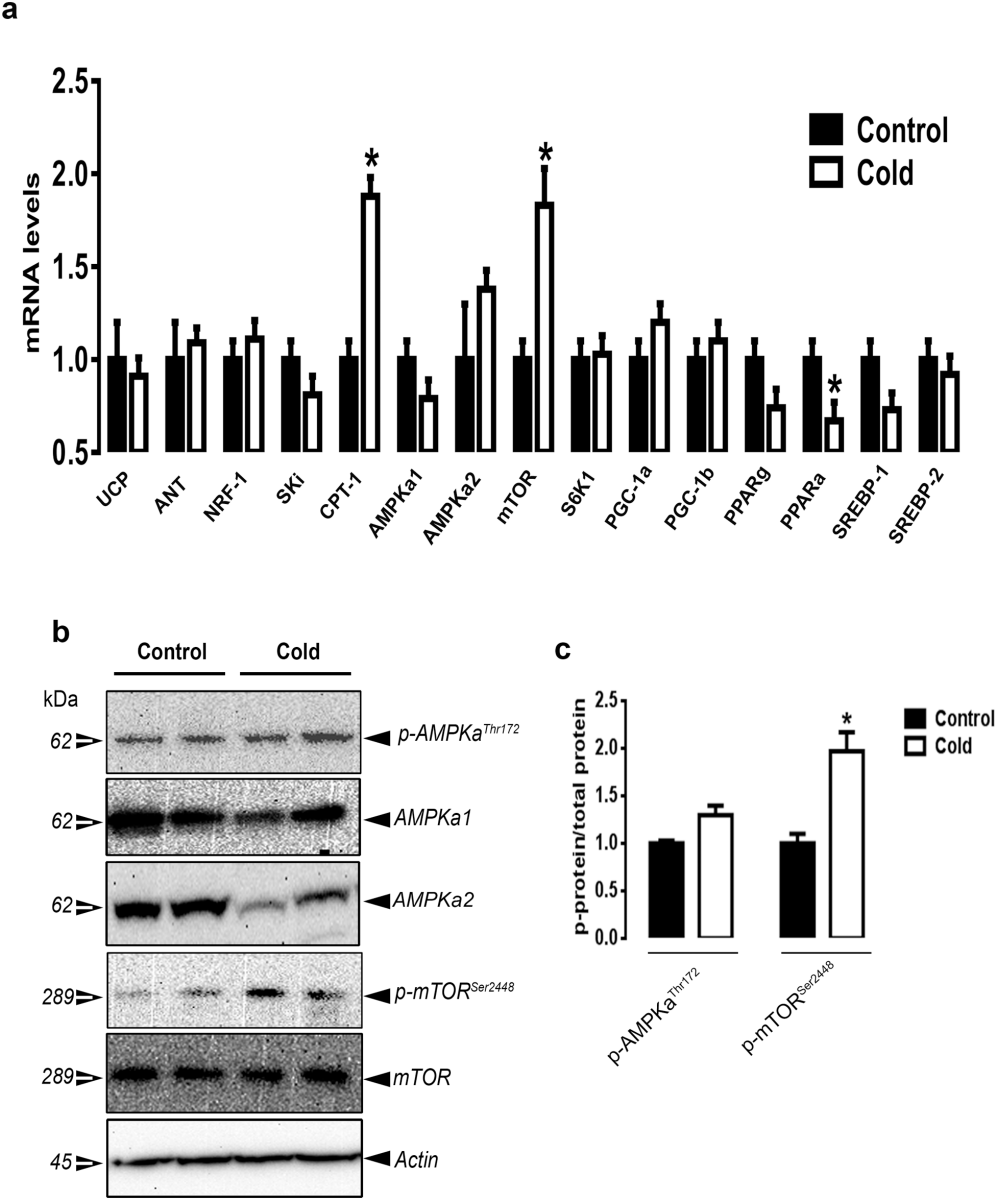
Effect of CMCC on metabolic-related genes in muscle of young broiler chicks. Relative expression of mitochondrial- and metabolic-related genes was determined by qPCR (a). Phosphorylated and total protein levels of AMPKα1/α2 and mTOR were determined by Western blot (b) and presented as p-protein/total protein ratio (c). The values represent the mean ±SEM (n = 6). * Indicates a significant difference between cold and control group (*P*<0.05).

**Fig 7:**
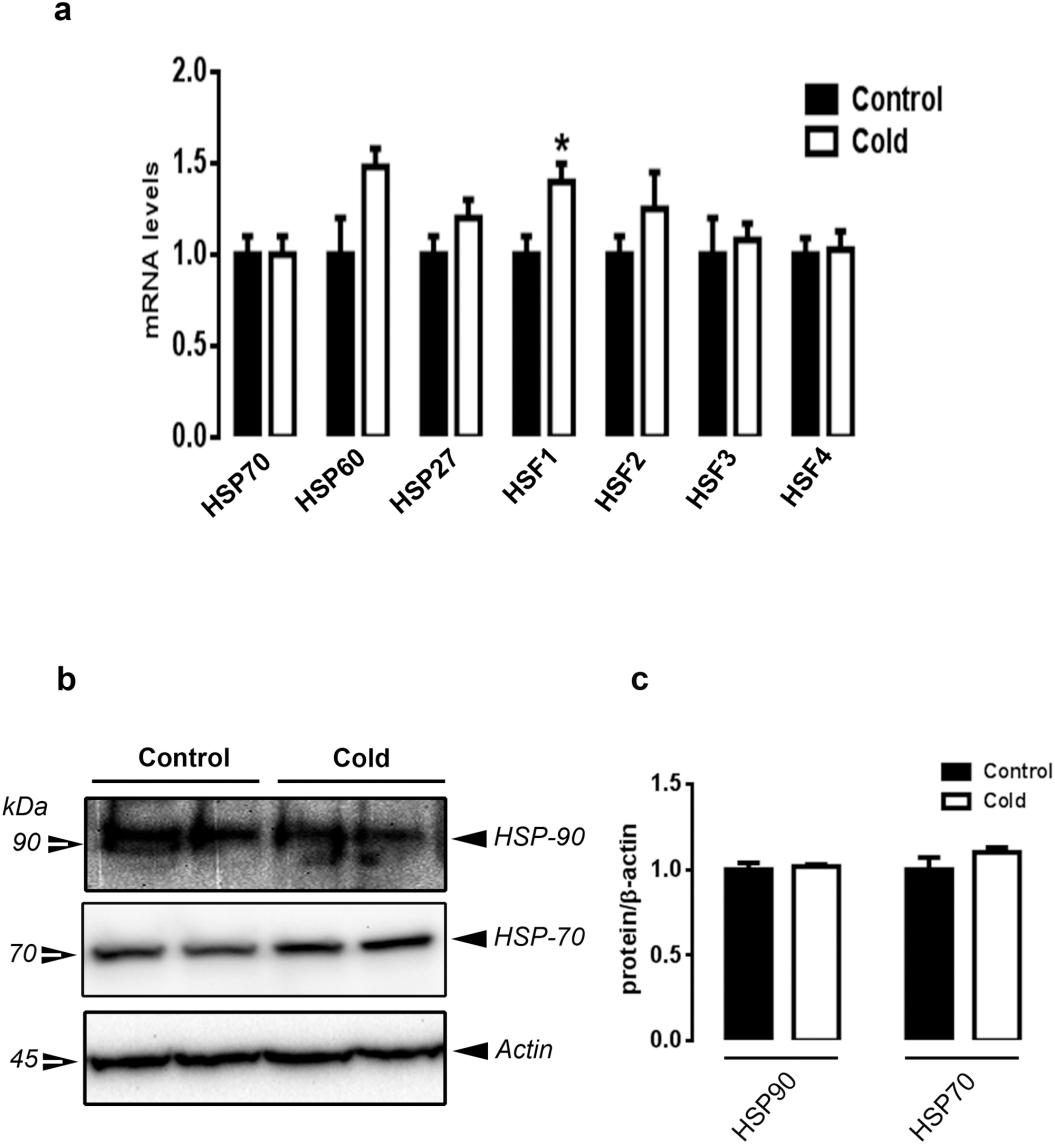
Effect of CMCC on HSP and HSF expression in the muscle of young broiler chicks. HSP and HSF mRNA levels were measured by qPCR (a). HSP70 and HSP90 protein levels were determined by Western blot (b) and presented as normalized ratio to β-actin (c). The values represent the mean ± SEM (n = 6). *Indicates a significant difference between cold and control group (*P*<0.05).

CMCC did not affect the expression of uncoupling protein (UCP), adenine nucleotide translocator (ANT), nuclear respiratory factor 1 (NRF-1) and the avian sarcoma viral oncogene homolog Ski, key genes involved in the regulation of mitochondrial function and energy expenditure (Fig 6a). However, it significantly induced the expression of carnitine palmitoyltransferase 1 (CPT-1), AMPKγ3 and mTOR and down-regulated the expression of peroxisome proliferator-activated receptor alpha (PPARα) compared to the control group (*P*<0.05, Fig 6a and S1 Fig). The expression of PPARγ, PPARγ coactivator alpha and beta (PGC-1α and PGC-1β), SREBP-1/2, AMPKα1/2, AMPKβ1/2, and AMPKγ1/2 did not differ between the two groups (Fig 6a and S1 Fig). In line with the variation of gene expression, CMCC increased the phosphorylated levels of mTOR^Ser2448^ compared to the control group (*P*<0.05, Fig 6b and 6c). Total AMPKα2 protein levels were significantly decreased in the CMCC compared to the control group (Fig 6b). Only HSF-1 gene expression was higher in CMCC chicks compared to the control group (P<0.05, Fig 7a); however the expression of HSP70, HSP60, HSP27, HSF2-4 did not differ between the two groups.

## Discussion

Low and high environmental temperatures are the most challenging stressors impacting both poultry and livestock industries. Cold stress, for instance, has been reported to cause an estimated total annual economic loss to the Chinese poultry industry of 100 million in currency [5]. A tremendous amount of seminal work has been done to identify the animal physiological and behavioral responses to cold stress and improve management strategies, however, the underlying molecular mechanisms are still not completely defined. In the present study, we aimed to determine the effect of CMCC on growth performance, plasma metabolite levels, and the expression of key metabolic-related genes.

The standard rearing practice in poultry industry is to maintain the average environmental temperature at 32°C during the first week of chick age and gradually decrease it (2 to 3°C/week) to about 22°C at 4 weeks. Although chickens are homeothermic and able to engage adaptive and/or protective mechanisms to cope with cold stress and re-establish their body temperature homeostasis, sudden severe cold stress could be detrimental. The strong negative effect of severe cold stress on avian growth performance, health and welfare is well documented [4,7,28,29]. In the present study, growth performances (BWG and FCR) were improved by CMCC during the first week and at the end of the experimental period (3 weeks) compared to the control group, corroborating previous studies in chickens exposed to short-term cold conditioning [6,30]. Shinder et al. [31] found that embryonic cold conditioning also improved growth performance and reduced ascites incidence in chickens. In contrast, Baarendse et al. [32] reported that exposure of chickens to a moderate reduction in house temperature during early post-hatching period have long-term negative effects on growth performance. The discrepancies between these findings might be related to several factors including chicken strain (Hubbard HY vs. Cobb chickens in Baarendse’s [32] and our studies, respectively), duration of cold exposure (5 vs. 7 days in Baarendse’s and our studies, respectively), and/or experimental conditions (diet composition, stress, density and feeding system).

In an attempt to better understand the mechanisms underlying the adaptation and acclimatization to CMCC, we measured circulating metabolic substrates in both chick groups. In agreement with previous studies in mammals and chickens [33–35], plasma CK and cholesterol levels were both higher in CMCC chicks compared to control group. CK, which catalyzes the reaction of ATP and creatine to phosphocreatine, is released into the circulation following changes in the permeability of the sarcolemma in response to various stressors [36] and is generally accepted as an indicator of muscle damage. As LDH levels were not affected during CMCC exposure in the present study, the increased levels of circulating CK indicates its potential role in maintaining high ATP turnover at low temperature as previously reported [37]. It is possible that the increased levels of plasma CK, observed in our study, is a result of an enhanced skeletal muscle mass [38,39] that is mirrored in higher BWG in CMCC compared to control chicks. Although the mechanisms underlying the CMCC-induced cholesterol elevation are not known at this time, we hypothesize that chicks heavily rely on mobilizing energy from body fat to satisfy their higher energy requirement.

As changes in growth performances (BWG and FCR) are mainly caused by feed intake, energy expenditure and intermediary metabolism, we next performed in depth analysis of key regulators gene expression in three metabolically important tissues; the brain (main site for feed intake regulation) [40], the liver (main site for lipogenesis) [41], and the muscle (main site for thermogenesis and energy expenditure) [42]. The brain contains the satiety and hunger centers and two separate populations of neuronal cell types. One synthesizes orexigenic neuropeptide Y (NPY) and agouti-related peptide (AgRP) [43,44], while the other produces anorexigenic pro-opiomelanocortin (POMC) and cocaine and amphetamine regulated transcript (CART) [45,46]. These neuropeptides interact in a complex way with each other and with the central melanocortin system, melanin-concentrating hormone, orexin, ghrelin and adiponectin, to mention a few, to regulate energy homeostasis in mammals [for review see [47]]. A recent study, using microarray analysis, reported 24h-cold exposure-induced changes in chicken hypothalamic gene expression [5]. Our work complements and adds to this study by showing that CMCC down regulated the expression of the potent orexigenic NPY and anorexigenic CART genes which may explain the similar feed intake observed between the control and CMCC chicks. Central adiponectin (Adpn) and its related receptors (AdipoR1 and AdipoR2) were down regulated by chronic CMCC in our experimental conditions. Although the role of adiponectin in the regulation of feed intake in mammals is controversial [48,49], its role has not been previously addressed in avian species. As we did not see an effect of CMCC on feed intake despite the significant decrease in central adiponectin system, we speculate that adiponectin might be involved in the regulation of thermogenesis, energy expenditure and/or lipid metabolism as previously reported in mammals [50]. We also identified central FAS-AMPK-mTOR as a new pathway involved in chronic mild cold response. This pathway has been reported to be involved not only in the regulation of energy homeostasis in both mammalian and avian species [51–54], but also in the regulation of energy expenditure [55] and lipid metabolism [56]. It is likely that during cold stress and in order to maintain their normal body temperature, the chicks increased their metabolic heat production by facultative/adaptive thermogenesis which in turn activates the key cellular energy sensor AMPK and thereby inhibits mTOR (another energy/nutrient-sensitive kinase) activation and the switch from anabolic to catabolic state.

Consistent with our hypothesis and in agreement with previous studies [57], CMCC down regulated the hepatic expression of the key lipogenic genes FAS (which catalyzes the synthesis of long-chain fatty acid synthesis through the condensation of acetyl-CoA and malonyl-CoA in a complex seven-step reaction), ACCα (multifunctional enzyme which catalyzes the carboxylation of acetyl-CoA to malonyl-CoA, the rate-limiting step in fatty acid synthesis) and ME (which catalyzes the oxidative decarboxylation of malate to pyruvate and generates NADPH for fatty acid biosynthesis). The concomitant decrease in hepatic SREBP1/2 mRNA abundance indicates that CMCC down regulated the lipogenic gene expression at transcriptional levels through these key transcriptional factors [58]. ACCα is also regulated at translational levels and by phosphorylation/dephosphorylation of targeted serine residues. The increase of ACCα phosphorylation at Ser^79^ indicates its inactivation by CMCC and further support the notion of fatty acid synthesis inhibition [59]. Furthermore, the decrease of hepatic mTOR phosphorylation consolidates the concept of activated catabolic pathways in the liver of chicks exposed to CMCC. mTOR has been found to play a role in lipogenesis with the finding that rapamycin (mTOR inhibitor) blocks the expression of lipogenic genes and impairs the nuclear accumulation of the SREBPs [60]. Although the exact molecular mechanisms by which SREBPs are regulated by mTOR are not well defined, it is believed to be mediated by ribosomal S6 kinase 1 (S6K1) [61]. As recently reported, mTOR may also regulate the SREBP-lipogenic gene transcriptional networks through the negative regulation of lipin 1 [62]. Several other molecular mediators such as PPARα/δ, Akt and PKA are not ruled out [63,64] and further studies are warranted.

Nonshivering thermogenesis (NST) is a common adaptive response to cold found in a number of mammalian and avian species [65], however the site and mechanisms appear to be different. While brown adipose tissue is well known to account for a large proportion of mammalian NST [66] via activation of UCP-1, skeletal muscle, however is the main site of avian NST [42]. Mechanisms involved in avian NST are still unclear but may involve reduced energetic coupling in skeletal muscle mitochondria through activation of an avian homolog of mammalian UCP1. We have previously identified avian muscle UCP [67] and we and others found that it was up regulated by short severe cold exposure [68–70]. Here, neither UCP nor other mitochondrial-related genes (ANT, NRF-1 and Ski) are altered by CMCC indicating that our experimental conditions are not harsh. The difference observed between this and previous studies may be related to severity and duration of environmental temperature at which birds were acclimated. Indeed, Duchamp’s group recently reported that muscle NST, UCP expression and the intensity of mitochondrial oxidative phosphorylation increased in proportion with the harshness of cold [69]. CMCC induced CPT-1, the rate-limiting enzyme in the β-oxidation of long chain fatty acids [71]. Taken together our data indicate that the decreased activity of ACCα causes a decrease in malonyl-CoA concentration which in turn induces CPT-1 activity and enhances fatty acid β-oxidation. The increased mRNA levels and phosphorylation of mTOR in the muscle of CMCC chicks indicate that mitochondrial fatty acid utilization and oxidation are very likely regulated by mTOR [72].

Finally to assess whether our experimental conditions were stressful or not, we determined the mRNA abundance and protein expression of HSPs, known as key stress markers, and their related transcription factors HSFs in all three tissues. During stress, HSPs are rapidly synthesized and are involved in folding/unfolding and assembly/disassembly of protein complexes to protect stressed cells. In our study, CMCC induced the expression of avian HSP-70 and HSP-27 only in the liver but not in the muscle or the brain, indicating a tissue-specific regulation of HSPs by cold exposure. Additionally, our data suggested that liver tissue may be more sensitive to cold stress compared to other tissues and supports a protective function of HSPs as previously reported in myocardium [20,73].

In summary, we provided new evidence that CMCC can improve growth performances (BWG and FCR) in young chicks through a modulation of key metabolic-related genes. AMPK and mTOR seem to be a key molecular signatures orchestrating cellular fatty acid utilization (inhibition of hepatic lipogenesis and increase of muscle fatty acid β-oxidation) to satisfy energy requirement during cold exposure. These findings open a new vista on the role of the AMPK-mTOR pathway in cold acclimation and further studies are needed to determine the up- and down-stream mediators that can be used in genetic selection to improve avian thermo (cold)-tolerance.

## Supporting Information

S1 Fig Effect of CMCC on AMPKβ and AMPKγ subunits in young broiler chickens. Relative expression of AMPKβ1/β2 and AMPKγ1/2/3 in the brain (a), liver (b), and muscle (c) was measured by qPCR using 2^-ΔΔCt^ method. Data are mean ± SEM (n = 6). * Indicate a significant difference between cold and the control group (*P*<0.05).
(TIF)
