# Supplementary figures and images for: Chronic Mild Cold Conditioning Modulates the Expression of Hypothalamic Neuropeptide and Intermediary Metabolic-Related Genes and Improves Growth Performances in Young Chicks
Source: PLoS One. 2015 Nov 16;10(11):e0142319. doi: 10.1371/journal.pone.0142319 (PMC4646505; doi:10.1371/journal.pone.0142319)

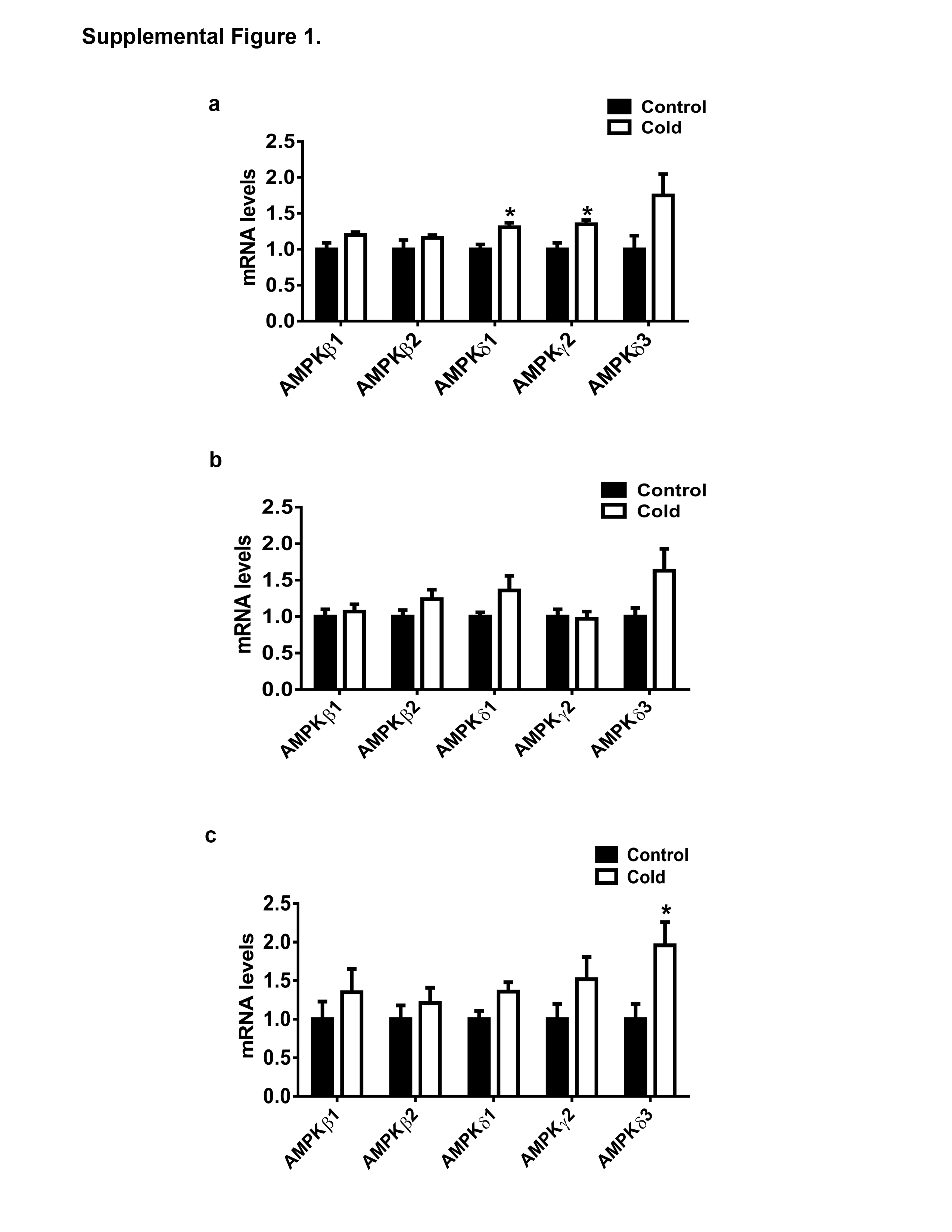

Supplement: S1 Fig — Relative expression of AMPKβ1/β2 and AMPKγ1/2/3 in the brain (a), liver (b), and muscle (c) was measured by qPCR using 2-ΔΔCt method. Data are mean ± SEM (n = 6). * Indicate a significant difference between cold and the control group (P<0.05). (TIF) [file pone.0142319.s001.tif]
